# On the Toxicity of Therapeutically Used Nanoparticles: An Overview

**DOI:** 10.1155/2009/754810

**Published:** 2009-01-25

**Authors:** A. El-Ansary, S. Al-Daihan

**Affiliations:** Biochemistry Department, Science College, King Saud University, P.O. Box 22452, Riyadh 11451, Saudi Arabia

## Abstract

Human beings have been exposed to airborne nanosized particles throughout their evolutionary stages, and such exposures have increased dramatically over the last century. The rapidly developing field of nanotechnology will result in new sources of this exposure, through inhalation, ingestion, and injection. Although nanomaterials are currently being widely used in modern technology, there is a serious lack of information concerning the human health and environmental implications of manufactured nanomaterials. Since these are relatively new particles, it is necessary to investigate their toxicological behavior. The objective of this review was to trace the cellular response to nanosized particle exposure. Therapeutic application of selected nanoparticles together with their range of toxic doses was also reviewed. Effect of therapeutically used nanoparticles on cell membrane, mitochondrial function, prooxidant/antioxidant status, enzyme leakage, DNA, and other biochemical endpoints was elucidated. This paper highlights the need for caution during the use and disposal of such manufactured nanomaterials to prevent unintended environmental impacts.

## 1. Introduction

Nanotechnology involves the creation and manipulation of materials at nanoscale levels (1–100 nm) to create products that exhibit novel properties [1]. The application of nanotechnology to medicine, known as nanomedicine, concerns the use of precisely engineered materials at this length scale to develop novel therapeutic and diagnostic modalities [2]. Nanomaterials have unique physicochemical properties, such as ultra small size, large surface area to mass ratio, and high reactivity, which are different from bulk materials (in microscale) of the same composition. These properties can be used to overcome some of the limitations found in traditional therapeutic and diagnostic agents. Nanotoxicology is emerging as an important subdiscipline of nanotechnology. Nanotoxicology refers to the study of the interactions of nanostructures with biological systems with an emphasis on elucidating the relationship between the physical and chemical properties (e.g., size, shape, surface chemistry, composition, and aggregation) of nanostructures with induction of toxic biological responses [3, 4].

Many people can get exposed to nanostructures in a variety of manners such as researchers manufacturing the nanostructures, patients injected with nanostructures, or people using products containing nanostructures. In all cases, there will be unique routes of exposure that will dictate the specific fate of nanostructures. The overall behavior of nanostructures could be summed as follows: (1) nanostructures can enter the body via six principle routes: intra venous, dermal, subcutaneous, inhalation, intraperitoneal, and oral [5]; (2) absorption can occur where the nanostructures first interact with biological components (i.e., proteins, cells); (3) afterward, they can distribute to various organs in the body and may remain the same structurally, be modified, or metabolized [6]; (4) they enter the cells of the organ and reside in the cells for an unknown amount of time before leaving to move to other organs or to be excreted.

Recently, many studies focus on the safety issue of manufactured nanomaterials to minimize or eliminate their nanotoxicity even before they are widely used [3, 7–9]. This paper seeks to provide a comprehensive review of all articles published on toxicity of therapeutically used nanoparticles together with trials for modification of these products aiming to improve their biocompatibility and minimize their toxicity.

## 2. Nanomedicine

Nanomedicine has been defined as the monitoring, repair, construction, and control of human biological systems at the molecular level, using engineered nanodevices and nanostructures [10]. Our body is constructed from nanoscale building blocks such as DNA and proteins, which have long been targeted by the pharmaceutical industry long before the emergence of nanotechnology [11, 12]. This category of drugs includes aspirin, cisplatin, and other anticancer agents as well as much more complex molecules like beta-blockers and anti-inflammatory agents [13]. The difference between nanomedicine and conventional drugs is that nanomedicine is entirely based on small molecule chemistry. It not only covers the therapeutic agents themselves, but also promise to combine the abilities to deliver those agents to specific regions or tissues in the body, to specific cells, perhaps to a specific location within a cell, and also to make release of the therapeutic responsive to a physiological condition and perform specific task [14]. The increased biological activity of nanoparticles can be either positive or desirable (e.g., antioxidant activity, carrier capacity for therapeutic penetration of blood-brain barrier, and the stomach wall or tumor pores), dispersed throughout the whole body including entering the central nervous system, or negative and undesirable (e.g., toxicity, induction of oxidative stress, or cellular dysfunction) or a mix of both [3].

Nanoparticles have been found to be distributed to the colon, lungs, bone marrow, liver, spleen, and the lymphatics after intravenous injection [15]. Distribution is followed by rapid clearance from the systemic circulation, predominantly by action of the liver and splenic macrophages [16]. Clearance and opsonization, the process that prepares foreign materials to be more efficiently engulfed by macrophages, occur under certain conditions for nanoparticles depending on size and surface characteristics [16].

When inhaled, nanoparticles are found to be distributed to the lungs, liver, heart, spleen, and brain [15]. Nanoparticles are cleared in the alveolar region via phagocytosis by macrophages facilitated by chemotactic attraction of alveolar macrophages to the deposition site [17, 18]. The average half-life (*t* 1/2) for nanoparticles in the respiratory tract is ≈700 days in humans [3].

After intraperitoneal injection, nanoparticles have been found to cross the transplacental membrane or cross the peritoneal cavity into uterus. This affected the embryos cranial development and even caused embryo death [19].

After oral exposure, nanoparticles distribute to the kidneys, liver, spleen, lungs, brain, and the gastrointestinal (GI) tract [15]. Some nanoparticles can pass through the GI tract and are rapidly eliminated in feces and in urine, indicating that they can be absorbed across the GI barrier and into the systemic circulation [15]. However, some nanoparticle systems can accumulate in the liver during the first-pass metabolism [3].

Contact with nanoparticles through the skin can occur due to occupational exposure during the manufacturing of solvents, pesticides, or pharmaceuticals. Skin exposure to nanoparticles can also occur during nonoccupational situations from the use of cosmetics and in the intentional application of topical creams and other drug treatments [3].

### 2.1. Nanosized Drug

Many approaches have been developed to use nanoparticles in the area of biomedical imaging and drug delivery.Quantum dots (QDs), one of the most well studied, are nanocrystals that fluoresce in different colors depending on their sizes (e.g., cadmium selenide) [20]. They typically have a core made of inorganic element, but are generally coated with organic materials such as polyethylene glycol to enhance their biocompatibility or attach them to specific target molecule like proteins or DNA strands [21]. If the target molecule is an indicator of disease, detection of that molecule may indicate a higher propensity for disease. An example is to use nanoparticles to bind to blood clots and to helpmake clots more visible by ultrasound [22].

Nanoshells, another nanodelivery system that is composed of copolymers, are used in combination with specific wavelengths of light and heat technology for cancer therapy. Gold nanoshells (GNSs) are particularly suitable for use in the surgical arena as their outer shell is composed of a commonly used reduced inert gold. When activated by near infrared light, GNS can raise surrounding temperatures to levels sufficient for cellular ablation. This strategy was recently used by Stern and Cadeddu [23], for the therapeutic ablation of urologic malignancies.

Incorporation of cancer-killer genes into nanocapsules is being tried out. One of the genes being investigated is the gene elaborate tumor necrosis factor, a protein that is toxic not only to cancer but also to healthy cells when injected in large doses. To avoid damage to normal tissue, the nanocapsule is coated with sensors that target only on tumor cells.

Delivery of drugs to the brain has always been a challenge. The use of nanoparticles to deliver drug to the brain using the pathfinder technology is being investigated. This technology uses nanoparticulate drug carriers in combination with the novel targeting principle of “differential protein adsorption” to cross the blood-brain barrier [24]. As the nanoparticles are not efficiently scavenged by macrophages, the resulting increase in blood circulation time and hence bioavailability are expected to extend the duration of controlled system drug delivery or to improve the prospects for nanoparticles to reach target sites by extravasation [25].

The delivery of magnetic nanoparticles (MNPs) to or into various cell types has become an area of increasing interest in the biomedical sciences [26, 27]. Targeted delivery is used to deliver drugs or genes by attaching them to MNPs and locally concentrating the resulting complexes in vivo to the desired locale [28]. Similarly, magnetic hyperthermia, the local concentration of MNPs and subsequent heating via magnetic fields, has shown promise as a potentially viable cancer therapy [29].

Nanosized calcium fluoride (CaF_2_) that could be used as F reservoir for more effective F regimens was recently prepared [30]. The nano-CaF_2_ can be used as an effective anticaries agent in increasing the labile F concentration in oral fluid and thus enhances the tooth remineralization. It can also be very useful in the treatment for the reduction of dentin permeability.

Nanomedical research could result in an array of new medical devices. Interesting research projects include use of nanoelectromechanical device or nanowire field-effect transistor to detect insect baculovirus and single influenza viruses, respectively, were conducted [31]. It is hoped that development of these nanodevices can help physician to locate the problem areas in the body more precisely. Other research works involve the use of biochips and microfluidic devices to screen tissues for genetic differences and to design genetically targeted drugs [32].

## 3. Nanotoxicology

While the small size of particles is what makes nanotechnology so useful in medicine and industry, it is also one of the main factors that might make them potentially dangerous to human health. Research is now showing that harmless bulk materials opinion is that the smaller the particles are the more reactive and toxic are their effects.It is because any intrinsic properties of particles will likely be emphasized with the increase in surface area per unit mass [33, 34].

The potential risks inherent to any new technology are recognized. However, the special concern with nanotechnology is the unique type of toxicity due to surface modification. Enhanced endocytosis including a potential for inflammatory and prooxidant activity are shown to be largely dependent on nanoparticles' surface chemistry (coating) and in vivo surface modifications [3, 33]. Increase in pulmonary toxicity (e.g., inflammation, granuloma formation) of carbon nanotubes when compared with that of the carbon black and carbonyl iron particles was seen in mice and rats [7, 34].

Oxidative stress caused by free radicals generated by the interaction of particles with cells may result in cell death. Evidence of mitochondrial distribution and oxidative stress response after endocytosis of nanoparticles was noted. It was suggested that nanoparticles, because of their small sizes, could act like haptens to modify protein structures, either by altering their function or rendering them antigenic, thus raising their potential for autoimmune effects [35].

There is an initial finding that indicated that gold nanoparticles might move through a mother's placenta to the fetus [36]. Efficient uptake of nanoparticles via the gastrointestinal tract has also been well documented in oral feeding studies and gavage studies [37]. All these findings indicate that nanoparticles may potentially present problems with body burdens and it is hypothesized that nanoparticles, because of their long retention in the body tissues, might repeat their highly catalytic activity with the host in cascade [38].

Though nanosilver-based dressing and surgical sutures have received approval for clinical application, and good control of wound infection is achieved, their dermal toxicity is still a topic of concern. Despite laboratory and clinical studies confirming the dermal biocompatibility of nanosilver-based dressings, several other researchers have demonstrated the cytotoxicity of these materials [39–44]. Paddle-Ledinek et al. [45] exposed cultured keratinocytes to extracts of several types of silver containing dressings. The results showed that extracts of nanocrystalline coated dressings are among those, which are the most cytotoxic. Keratinocyte proliferation was significantly inhibited, and cell morphology was affected [45].

Recently, the identification of cytotoxicity of nanoparticles toward mammalian germline stem cells has aroused great concern over the biosafety of nanomaterials [46]. In their study, they used a cell line with spermatogonial stem cell characteristics to test in vitro toxicity of several types of nanoparticles. The results showed that of all the tested materials (Ag, MoO_3_, and Al), silver nanoparticles were the most toxic with manifestations like drastic reduction of mitochondrial function, increased membrane leakage, necrosis, and induction of apoptosis. The findings are of significant practical implications because silver nanoparticles are now able to access human sperms via a variety of commercialized products like contraceptive devices and maternal hygiene items. Based on this, fertility problems may occur. In addition, as a fair extrapolation, another question emerged: what they will do to egg cells?

Liver appears to be a major accumulation site of circulatory silver nanoparticles [47]. Like germ line stem cells, similar patterns of cytotoxicity of silver nanoparticles (decrease of mitochondrial function, LDH leakage, and abnormal cell morphologies) were observed with in vitro BRL 3A rat liver cells, but to a lesser extent [1]. In another study by the same researchers, a neuroendocrine cell line (PC-12 cells) was exposed to silver nanoparticles as a control against Mn nanoparticles and Mn^2+^ [48]. Experimental results showed that silver nanoparticles were toxic to mitochondria than to Mn and Mn^2+^.

All of these findings are of importance because considerable amount of silver could be detected in rat brain following inhalation of silver nanoparticles [47]. The neurological toxicity of silver is not clinically ascertained, however, several seizures cases have been related to exposure to silver or silver compounds [49]. Thus, mitochondria seem to be sensitive targets of cytotoxicity of silver nanoparticles. However, the mechanism of silver nanoparticles action on mitochondria is yet to be elucidated. In the study, with BRL 3A liver cell line, depletion of glutathione (GSH) level and increased ROS were found in association with mitochondrial perturbation, suggesting that oxidative stress might mediate the cytotoxicity of silver nanoparticles. Based on these findings, a preliminary impression can be formed that silver nanoparticles may interact with proteins and enzymes with thiol groups within mammalian cells. These proteins and enzymes like glutathione, thioredoxin, SOD, and thioredoxin peroxidase are key components of the cell's antioxidant defense mechanism which is responsible to neutralize the oxidative stress of ROS largely generated by mitochondrial energy metabolism. As these effects of Ag^+^ could be completely blocked by sulfhydryl reagents as GSH, the surface modification of silver nanoparticles by phosphoryl disulfides was effective in improving silver biocompatibility and intracellular uptake [50]. They prepared the phospholipid derivatives containing disulfide groups to modify silver nanoparticle surfaces. By adding sodium borohydride to reduce both disulfide bonds of the derivatives and silver ion simultaneously, the generated thiol group can be reacted with newborn silver atoms immediately to generate nanoclusters. The assemblies consisted of either phosphorylcholine (PC) or Phosphorylethanolamine (PE) head groups, which made the silver clusters biocompatibile. In cell culture tests, the surfaces modified nanoparticles were internalized into platelet and fibroblast cells in a short period of incubation without harming the cells.

**Figure 1:**
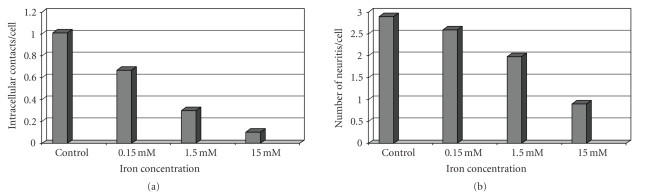
(a) Number of intracellular contacts and (b) formation of neuritis in nano-Fe_2_O_3_ treated PC12 nerve cells.

The study of Pisanic II et al. [51] indicates that even temporary exposure to Fe_2_O_3_ magnetic nanoparticles (MNPs) results in a dose-dependent reduced ability rat pheochromocytoma growing neuron cell line PC12 to respond to nerve growth factor (NGF). PC12 cells exposed to different doses of Fe_2_O_3_ MNPs show reduced viabilities, increased cytoskeletal disruption, decreased intracellular contact, and diminished ability to form mature neuritis in response to NGF exposure as compared to control cells (Figure 1). This may have significant implication for in vivo phenotypic dependent in vitro uses of Fe_2_O_3_ in general. Wiwanitkit et al. [52] demonstrated in a preliminary small study that the motility of spermatozoa was affected by the presence of gold nanoparticles. They noted that gold particles can penetrate sperm cells, which could result in fragmentation. The possible spermatotoxicity of gold in industrial use has been reported elsewhere as a cause of male sterility [53].

### 3.1. Ultrahigh Reactivity Provokes Nanotoxicity

**Figure 2:**
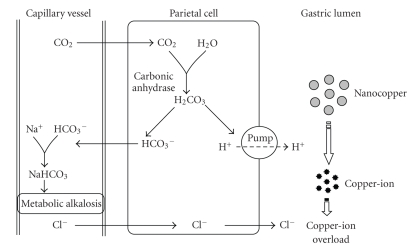
Mechanism of metabolic alkalosis induced by nanocopper ion.

Because the nanosize/surface area of the nanosubstance is directly correlated to many essential characteristics like surface properties, chemical reactivity, physical absorption ability, and so forth, all these factors strongly dominate nanotoxicological behavior in vivo [54]. The study of Chen et al. [43] on the acute oral toxicity of copper particles has shown a significant correlation with its size distribution. With the particle size reducing from 17 *μ*m (particle number: 44 per *μ*g; surface area 3.99 × 10^2^  cm^2^/g) to 23.5 nm (particle number: 1.7 × 10^10^ per *μ*g; surface area 2.95 × 10^2^  cm^2^/g), LD_50_ of copper particle sharply increases from >5000 mg/kg (nontoxic) to 413 mg/kg (moderately toxic) based on the Hodge and Sterner scale. For identical chemical composition, why do the nanocopper particles possess unique biobehavior (nanotoxicity) in vivo comparing to those in bigger size (in microscale)? Looking for answer for this question may provide an insight into nanotoxicity reducing or elimination. They explained this on the basis that nanocopper particles may not compromise the mice directly. The nanocopper retained in gastric lumen can continuously react with the secreted acid juice. The depletion of H^+^ results in metabolic alkalosis because the HCO_3_ generated during production of gastric acid will return to the circulation resulting in the formation of large quantities of sodium bicarbonate which increase the arterial blood pH (Figure 2).

The elevation of blood pH motivates a set of compensatory effects: (a) a respiratory compensation which is limited [55], (b) renal compensation starts relatively later but can sustain for a long time (several days) [55, 56]. However, a series of abnormalities were found in the pathological examination such as swollen glomerulus, dwindling in lumen Bowman's capsules, and being signs of glomerulonephritis. The renal dysfunction may largely weaken renal compensation in nanocopper group and deteriorate the metabolic alkalosis.

It is reported that copper ions ingested are metabolized in liver and excreted via urine [57]. If the intake of copper exceeds the range of the tolerance, it would cause toxic effects to hepatic and renal tissues, which is consistent with the finding of Meng et al. [58] that nanocopper possesses extremely high bioavailability, hence, the original safety limit may be modified to much lower level. Based on these finding, we can suggest that nano-and microcopper exhibit different biological behaviors in vivo via oral exposure routine. In terms of nanocopper particle, both copper overload and metabolic alkalosis contribute to their grave toxicity.

High chemical reactivity of Ag nanoparticles was observed in the reaction with hydrochloric acid: Ag (nanoparticles) + HCl → AgCl + H_2_; the reaction product silver chloride was characterized by X-ray powder diffraction to give a direct evidence for the reaction which has been proved impossible for the bulk Ag [59].

The microscale titanium dioxide (TiO_2_), widely used in pharmaceutical and cosmetics industries, is considered as biologically inert [60]. Such that, there was no obvious lung toxicity in rats when a single instilled dose of TiO_2_ was 5 mg/rat or 50 mg/kg [61]. However, many studies have demonstrated that when TiO_2_ particles size decreased to nanoscale dimension, they could produce more pulmonary toxicity than their bulk counterparts [62–65]. In a comparative study done by Li et al. [66], the acute pulmonary toxicity induced by 3 and 20 nm TiO_2_ was investigated through measurement of selected biochemical parameters in bronchoalveolar lavage fluid (BALF). At 3-day postexposure, the 3 nm TiO_2_ induced significant increase of albumin, alkaline phosphatase (ALP), and acid phosphatase (ACP) concentrations in high-dose group (40 mg/kg) and also induced significant increase of ALP and ACP concentrations in mid-dose group (4 mg/kg), but did not induce significant increase of total protein and LDH concentrations in any dose group. On the other hand, 20 nm TiO_2_ induced significant increase of all biochemical parameters in high- and mid-dose groups. At 3-day postexposure, both TiO_2_ particles did not induce obvious pulmonary toxicity in their low-dose (0.4 mg/kg) groups as evidence of no significant increase of all biochemical parameters. The pH values of 3 nm TiO_2_ particles colloid were 5.38 ± 0.12, 4.55 ± 0.07, and 4.42 ± 0.13 at concentrations of 0.1, 1, and 10 mg/mL, respectively, while at the corresponding concentration, the pH values of 20 nm TiO_2_ particles suspension were5.5 ± 0.19, 4.64 ± 0.11, and 3.75 ± 0.04, respectively. They reported that pH value of TiO_2_ particles in medium, other than particle size, surface area, and aggregation, plays important role in affecting TiO_2_ nanoparticles pulmonary toxicity.

The potentiated toxicity of nanoscale vanadium oxide (V_2_O_3_) compared to bulk material is demonstrated in human endo- and epithelial lung cells and might be due to the higher catalytic surface of the particles [60]. Reduction in cell viability is almost ten times stronger and starts with the lowest concentrations of “nanoscaled” material (10 *μ*g/mL). Vanadium oxide leads to an induction of heme oxygenase 1 (HO-1) in a dose-dependent manner in ECV304 cells, whereas a reduction in protein levels can be observed for the epithelial cells (A549). Lipid peroxidation can be observed also for “nanoscaled” vanadium oxide to a much stronger extent in macrophages (RAW cells) than for bulk material. The observed effects cannot only be explained by oxidation from V_2_O_3_ to V_2_O_5_ as there are significant differences between the novel nano-vanadium and all used bulk materials (V_2_O_3_ and V_2_O_5_). It appears rather to be a nanoeffect of a high surface reactivity, here coupled with a yet unknown toxicity potentiating effect of a technically important catalyst.

## 4. Protein-Nanoparticle Interactions

Within the medical device community, it is now well accepted that material surfaces are modified by the adsorption of biomolecules such as proteins in a biological environment [67, 68], and there is some consensus that cellular responses to materials in a biological medium reflect the adsorbed biomolecule layer, rather than the material itself. However, the importance of the adsorbed protein layer in mediating interactions with living systems has been slower to emerge in the case of nanoparticle-protein interactions. The key role of protein-nanoparticle interactions in nanomedicine and nanotoxicity has begun to emerge recently with the development of the idea of the nanoparticle-protein “corona”. This dynamic layer of proteins (and other biomolecules) adsorbs to nanoparticle surfaces immediately upon contact with living systems. The composition of the protein corona at any given time will be determined by the concentrations of the over 3700 proteins in plasma [69] and the kinetic on and off rates (or equilibrium binding constants) of each protein for the particular nanoparticle. This corona may not immediately reach equilibrium when exposed to a biological fluid. Proteins with high concentrations and high association rate constants will initially occupy the nanoparticle surface, but may also dissociate quickly to be replaced by proteins of lower concentration, slower exchange, and higher affinity. Thus, the protein corona is the biological identity of a nanoparticle, as it is what the cell “sees” and interacts with. Functional changes of proteins of such complexes may be another mechanism by which particularly small nanoparticle, with their large surface area as a binding interface, may induce protein mal-functioning, which may lead to the pathogenesis and adverse health effects [70]. Survey of the literature on nanoparticle-protein binding shows that the vast majority of nanoparticle types studied, so far, bind apolipoproteins [68]. At first sight, this is surprising result and quite distinct from that for a flat surface. However, the fact that apolipoproteins are known to be involved in lipoprotein complexes, which themselves have sizes on the nanoscale ranging from 100 nm (chylomicron) to 10 nm (high-density lipoprotein), may mean that there are specific size-dependent interactions that drive the binding of apolipoprotein to nanoparticles. This is interesting from the point of view of nanoparticle interaction with cells, as lipoprotein complexes are involved in the general cellular processes of cholesterol metabolism [2]. Thus, there are multiple receptors for apolipoprotein complexes at cell surfaces that nanoparticles with surface-adsorbed apolipoproteins can potentially exploit to enter cells [71]. If we consider the issues of nanoparticle transport and fate in animals and humans, then it is also relevant that apolipoprotein E has been found to associate to some nanoparticles [67]. This has potentially significant consequences for neurotoxicity and the development of neurotherapies, as apolipoprotein E is known to be involved in trafficking to the brain [71].

The first reports of the direct biological influence of proteins adsorbed to nanoparticles are now emerging. Single-walled carbon nanotubes (SWNTs) and 10 nm amorphous silica coated with albumin have been shown to induce anti-inflammatory responses in macrophages, measured as inhibited induction of cyclooxygenase-2- (Cox-2) by lipopolysaccharide under serum-free conditions [72]. Blocking the adsorption of albumin by precoating the nanoparticles with nonionic surfactant (Pluronic F127) also inhibits the anti-inflammatory properties of the nanoparticles. These observations suggest an important role for the adsorbed proteins in modulating the uptake and toxicity of SWNTs and nanosized amorphous silica [72]. However, as these studies were conducted under serum-free conditions, it is unclear whether the albumin would remain bound to nanoparticles under competitive binding conditions, such as those occur in plasma or in a cellular milieu. The interaction between human adult hemoglobin (Hb) and bare CdS QDs dramatically alters the conformation of Hb and decreasing the *α*-helix content of the secondary structure from 72.5% to 60.8%. Raman spectra results indicate that the sulfur atoms of the cysteine residues form direct bonds on the surface of the CdS QDs [73]. Functionalization of nanoparticles surfaces with peptides is increasingly being used to control the interaction of nanoparticles with proteins [69].

## 5. Discussion

Nanotoxicology refers to the biokinetic evaluation of engineered nanostructures and nanodevices. The need for this area of investigations became apparent after the intensive expansion of nanotechnology, which in the last two decades has been widely used in the pharmaceutical industry, medicine, and engineering technology [74, 75]. Particle toxicology and the consequent adverse health effects of asbestos fibers and coal dust serve as a historical reference points to the development of nanotoxicological concepts.

**Figure 3:**
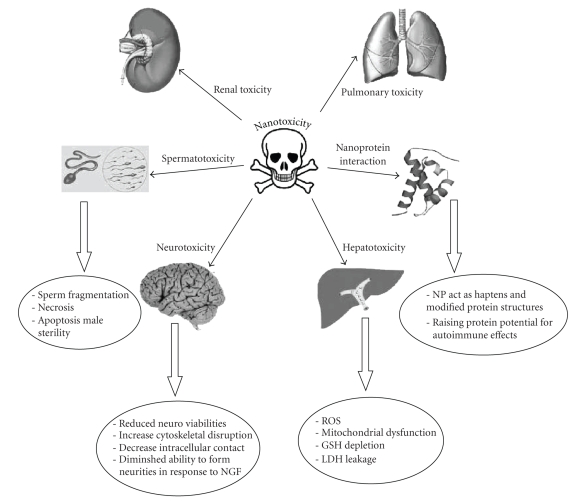
A summary of the most important recorded toxic effects of therapeutically used nanoparticles reviewed in the present paper.

In the area of medicine, nanomedicine has been defined as the monitoring, repair, construction, and control of human biological systems at the molecular level, using engineered nanodevices and nanostructures [10]. Macrophages as specialized host defense cells, endothelium as thin specialized epithelial cells that line the inner surface of lymph vessels and blood vessels serve as gate keeper to control passage of materials together, and tumors are the most common targets of nanoparticles. Within these biological targets, nanoparticles favor the formation of prooxidants especially under exposure to light, ultraviolet light, or transition metals; thereby destabilizing the balance between the production of reactive oxygen species (ROS) and the biological system's ability to detoxify or repair the system [74, 75]. ROS can also be produced by the NADPH oxidase in phagocytic cells as target of nanoparticle devices. Nanoparticles can modify mitochondrial function as well as cellular redox signaling. Oxidative stress induced by nanoparticles is reported to enhance inflammation through upregulation of redox-sensitive transcription factors including nuclear factor kappa B (NFkB), activating protein (AP-1), and extracellular signal regulator kinases (ERK) C-Jun, N-terminal kinases JNK, and p38 mitogen-activated protein kinases pathways. Figure 3  summarizes the most important recorded toxic effects of therapeutically used nanoparticles reviewed in the present paper.

## 6. Concluding Remarks

(1) Nanotechnology is growing at an exponential rate and will undoubtedly have both beneficial and toxicological impact and consequences on health and environment.

(2) As a result of their properties, nanomaterials differ substantially from those bulk materials of the same composition,allowing them to perform exceptional feats of conductivity,reactivity, and optical sensitivity. Possible undesirable resultsof these capabilities are harmful interactions with biologicalsystems with the potential to generatetoxicity.

(3) Development of new techniques to show accurate correlations between in vitro and in vivo studies is imperative to accurately portray nanoparticle effects. Moreover, toxicity studies are critical to establish the full in vivo potential of nanomedicine. Understanding the physiochemical, molecular, and physiological processes of nanoparticles is important for nanomedicine to become a reliable and sustainable treatment modality.

(4) In the future, nanoparticles could be classified in terms of their biomolecule corona which mediates their interaction with cellular machinery. This would represent a truly new paradigm in the field of nanoscale toxicology and in the design of safe nanocarriers for nanomedicine.

(5) With this new opportunity to utilize the unique properties of nanoparticles for research, industry, and medicine, there is a responsibility to test and optimize these new nanomaterials early during the development process to eliminate or ameliorate identified toxic characteristics.

(6) The rapid commercialization of nanoparticles requires focused environmental, health, and safety research, meaningful and open discussion of broader societal impacts and urgent oversight.
